# Annotations

**Published:** 1905-07-22

**Authors:** 


### ANNOTATIONS.

### The Hours of Sleep.

In his paper dealing with the hours of sleep at public schools Dr. T. D. Acland has raised the discussion of a very important question. There can be no doubt that the amount of sleep taken in the 24 hours has an important influence on health and efficiency, and this, more particularly, in the early and strenuous days of active youth. Doubtless it must be allowed that habit, here as elsewhere, may do something both in one direction and the other, but it is equally true that a point may be reached where even this influence is powerless. It is not unimportant to suggest that in attempting to prescribe the amount of sleep necessary or advisable for any individual considerable regard ought to be paid to the question of idiosyncrasy. Numerous rules, reading for the most part with a somewhat proverbial twang, have been framed on this subject for the guidance and encouragement of mankind, but there is no evidence that the authors of these wise saws had any basis for their suggestions outside the limits of their personal experiences. And it is because of this that their recommendations are essentially at fault. The practice of medicine affords numerous opportunities of studying the influence of various habits upon health and activity, and we may venture to suggest that the position of sleep in this respect is worthy of more attention than it commonly receives. We believe it will be found that the requirements of individuals in regard to sleep vary very widely, and that no hard-and-fast rule can be or ought to be defined. Rules of this order already exist, and are, we believe, harmful rather than helpful. Ambitious and diligent youths are introduced to examples of some few exceptional men who accomplished great ends by the sacrifice of hours usually devoted to sleep, and in the effort to emulate these examples cultivate studies at late hours as a proof of virtue and a guarantee of future distinction. The result is often disastrous, as might, indeed, have been expected. Most persons of intelligence find out by experience what habits are necessary to maintain at a maximum their physical and mental vigour. But when experience is yet to gain it is easy to be misled by false examples and unreliable copy-book maxims.

### Cremation in Scotland.

The Scottish Burial Reform and Cremation Society, to judge from its thirteenth annual report, is not able to claim the success of any large influence on public opinion. The number of cremations carried out at Maryhill during the last year was 22, which is an increase of four over the previous year. These figures show, what indeed was to be expected, that sentiment, custom, and prejudice are factors which demand for their removal a long and persevering educational process. An illustration of the force of these influences can be found in the indifference with which disregard of the wishes of the dying on this point is viewed. It is well known that within the last few years two distinguished Scottish noblemen left precise directions that their bodies were to be cremated. Yet their wishes were neglected by the surviving relatives, and this conduct, which in other directions would have been considered shameful, was apparently approved by public opinion. In spite, however, of discouragement the Scottish Society, we are glad to see, continues to advocate cremation as the most sanitary method of disposing of dead bodies, and it is not without influential support. At the last annual meeting of the society two distinguished medical sanitarians appeared on the platform and spoke energetically in favour of cremation as opposed to earth burial. Sir Henry Littlejohn related his experience in investigating the condition of some of the Edinburgh cemeteries and churchyards, and described the condition of matters there existing as of great social importance. Cremation he urged as advantageous on many grounds, and not the least because it demanded inspection of the body by two medical practitioners, and so diminished the chance of foul play. Dr. Ebenezer Duncan also urged that continued efforts should be made to influence public opinion. The popular notion that in cremation the body was put into a furnace was as injurious to the society as it was incorrect. The truth was that the body was consumed by heated gases, and the process was quite free from anything that could offend the most sensitive mind.

### Enteric Fever and Shell-fish.

Some interesting observations relative to shell-fish as a possible means of communication of enteric fever, are recorded by Dr. A. K. Chalmers in his annual report on the public health of the city of Glasgow. It has been noticed in several previous summers that there have been a number of outbreaks of enteric fever among the visitors at certain coast resorts, and that there has been reason to believe that these were associated with the consumption of shell-fish. At the same time the distribution of shell-fish from these centres has not appeared to have any prejudicial influence, and this contrast has suggested the advisability of an inquiry with a view to the discovery, if possible, of an explanation. Observation on the spot has shown that gatherers of shell-fish on the shores of the Gareloch, whence large quantities are despatched to the Glasgow market, are in the habit of avoiding those parts of the shore which receive sewage from neighbouring dwellings, being alive apparently to the business risks which may be incurred should an outbreak of infectious disease follow the consumption of their market products. On the other hand, visitors, it was found, frequently gathered mussels from areas subject to sewage pollution, being ignorant either of the fact of pollution or of the risks attached to it. Further, in some instances it has been observed that whilst the regular gatherers rejected certain specimens which they regarded as "sick," visitors, having less experience, and therefore less powers of discrimination, would gather and consume such specimens without hesitation. These observations go far to explain the contrast above noted. They also suggest that steps should be taken at the various coast resorts to warn visitors of the danger of using as food, shell-fish collected from the dangerous areas.

